# 2-Amino-6-methyl­pyridinium 4-nitro­benzoate

**DOI:** 10.1107/S1600536811003539

**Published:** 2011-02-05

**Authors:** Wei-Min Dai, He Zhou, Yi-Qiao Hu

**Affiliations:** a State Key Laboratory of Pharmaceutical Biotechnology, Nanjing University, Nanjing 210093, People’s Republic of China

## Abstract

In the crystal structure of the title salt, C_6_H_9_N_2_^+^·C_7_H_4_NO_4_^−^, the cations and anions are linked by N—H⋯O hydrogen bonds, forming chains running parallel to the *b* axis.

## Related literature

For background to ways of decreasing of bitterness in foods and medicines, see: Suzuki *et al.* (2002 ▶, 2004 ▶); Hofmann (1999 ▶); Shaw *et al.* (1984 ▶). For bond-length data, see: Allen *et al.* (1987 ▶). For related structures, see: Saminathan & Sivakumar (2007*a* ▶,*b* ▶); Näther *et al.* (1997 ▶); In *et al.* (1997 ▶); Harrison *et al.* (2007 ▶); Soriano-García *et al.* (1990 ▶); You *et al.* (2007 ▶). 
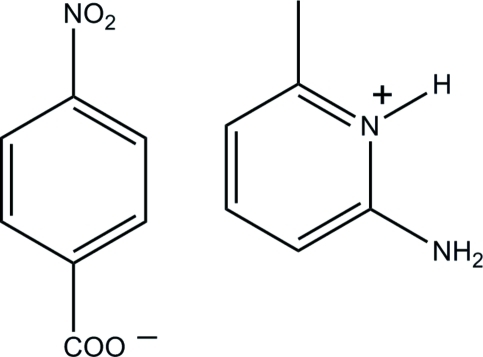


## Experimental

### 

#### Crystal data

C_6_H_9_N_2_^+^·C_7_H_4_NO_4_^−^
*M* *_r_* = 275.26
Monoclinic, 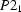

*a* = 8.0487 (11) Å
*b* = 6.7247 (9) Å
*c* = 12.7467 (17) Å
β = 101.802 (7)°
*V* = 675.33 (16) Å^3^
*Z* = 2
Mo *K*α radiation
μ = 0.10 mm^−1^
*T* = 298 K
0.20 × 0.20 × 0.18 mm

#### Data collection

Bruker SMART 1000 CCD area-detector diffractometer
Absorption correction: multi-scan (*SADABS*; Bruker, 2001 ▶) *T*_min_ = 0.980, *T*_max_ = 0.982
4175 measured reflections
1591 independent reflections
1265 reflections with *I* > 2σ(*I*)
*R*_int_ = 0.026

#### Refinement

*R*[*F* ^2^ > 2σ(*F* ^2^)] = 0.039
*wR*(*F* ^2^) = 0.105
*S* = 1.04
1591 reflections
191 parameters
5 restraints
H atoms treated by a mixture of independent and constrained refinement
Δρ_max_ = 0.12 e Å^−3^
Δρ_min_ = −0.17 e Å^−3^

### 

Data collection: *SMART* (Bruker, 2007 ▶); cell refinement: *SAINT* (Bruker, 2007 ▶); data reduction: *SAINT*; program(s) used to solve structure: *SHELXS97* (Sheldrick, 2008 ▶); program(s) used to refine structure: *SHELXL97* (Sheldrick, 2008 ▶); molecular graphics: *SHELXTL* (Sheldrick, 2008 ▶); software used to prepare material for publication: *SHELXTL*.

## Supplementary Material

Crystal structure: contains datablocks global, I. DOI: 10.1107/S1600536811003539/rz2548sup1.cif

Structure factors: contains datablocks I. DOI: 10.1107/S1600536811003539/rz2548Isup2.hkl

Additional supplementary materials:  crystallographic information; 3D view; checkCIF report

## Figures and Tables

**Table 1:** Hydrogen-bond geometry (Å, °)

*D*—H⋯*A*	*D*—H	H⋯*A*	*D*⋯*A*	*D*—H⋯*A*
N1—H1⋯O4^i^	0.91 (2)	1.75 (3)	2.649 (3)	173 (2)
N2—H2*A*⋯O3^ii^	0.89 (2)	1.94 (2)	2.812 (3)	170 (2)
N2—H2*B*⋯O3^i^	0.89 (2)	1.95 (2)	2.838 (3)	176 (2)

Symmetry codes: (i) 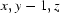; (ii) 
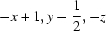
.

**Fig. 1.**
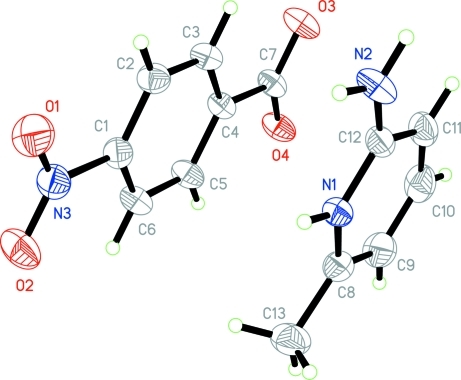
The structure of the title compound, showing 30% probability displacement ellipsoids and the atom-numbering scheme.

**Fig. 2.**
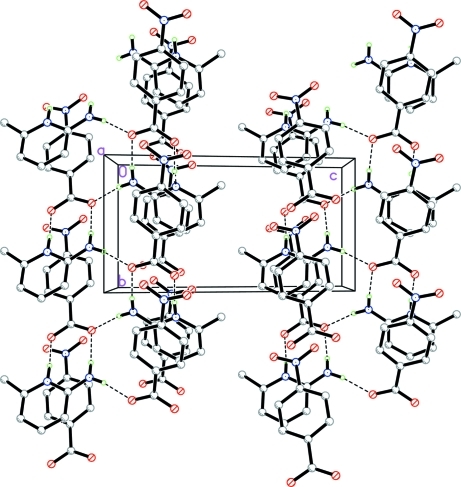
Crystal packing of the title compound, viewed along the b axis. Intermolecular hydrogen bonds are shown as dashed lines.

## supplementary crystallographic information

### Comment

Considerable attention has been recently paid to the decrease the bitterness of foods and medicines (Suzuki *et al.*, 2002; Suzuki *et al.*, 2004; Hofmann, 1999; Shaw *et al.*, 1984). 4-Nitrobenzoic acid is a bitter compound so, in order to investigate the influence of hydrogen bonds on its bitterness, the title compound was synthesized and its crystal structure is reported herein.

The asymmetric unit of the title salt consists of a 4-nitrobenzoate anion and a protonated 6-methyl-2-aminopyridinium cation (Fig. 1). The H atom of 4-nitrobenzoic acid is transferred to the N1 atom of 6-methyl-2-aminopyridine. All the bond lengths are within normal ranges (Allen *et al.*, 1987) and comparable with the values observed in similar compounds (Saminathan & Sivakumar, 2007*a*,*b*; Näther *et al.*, 1997; In *et al.*, 1997; Harrison *et al.*, 2007; Soriano-García *et al.*, 1990; You *et al.*, 2007). The C1—C6 benzene ring forms dihedral angles of 2.7 (2) and 0.2 (2)° with O1/N3/O2 and O3/C7/O4 planes, respectively. In the crystal structure (Fig. 2), intermolecular N—H···O hydrogen bonds (Table 1) link cations and anions into X-chains parallel to the *b* axis.

### Experimental

All the reagents used were of commercially grade and without further purification. 4-Nitrobenzoic acid (0.1 mmol, 16.7 mg) and 6-methyl-2-aminopyridine (0.1 mmol, 10.8 mg) were dissolved in MeOH/H_2_O (10 ml, 1:1 *v*/*v*). The mixture was stirred at room temperature for 30 min to give a clear colourless solution. After keeping the solution in air for 20 days, colorless block-shaped crystals were formed on slow evaporation of the solvents.

### Refinement

The amino H atoms were located in a difference Fourier map and refined isotropically, with the N—H and H···H distances restrained to 0.90 (1) and 1.45 (2) Å, respectively, and with *U*_iso_(H) set to 0.08 Å^2^. All other H atoms were placed in idealized positions and constrained to ride on their parent atoms with C—H distances of 0.93–0.96 Å, and with *U*_iso_(H) = 1.2*U*_eq_(C) or 1.5*U*_eq_(C) for methyl H atoms. In the absence of significant anomalous dispersion effects, Friedel pairs were averaged.

### Crystal data

**Table:**

C_6_H_9_N_2_^+^·C_7_H_4_NO_4_^−^	*F*(000) = 288
*M**_r_* = 275.26	*D*_x_ = 1.354 Mg m^−^^3^
Monoclinic, *P*2_1_	Mo *K*α radiation, λ = 0.71073 Å
Hall symbol: P 2yb	Cell parameters from 1260 reflections
*a* = 8.0487 (11) Å	θ = 2.5–24.5°
*b* = 6.7247 (9) Å	µ = 0.10 mm^−^^1^
*c* = 12.7467 (17) Å	*T* = 298 K
β = 101.802 (7)°	Block, colourless
*V* = 675.33 (16) Å^3^	0.20 × 0.20 × 0.18 mm
*Z* = 2	

### Data collection

**Table:**

Bruker SMART 1000 CCD area-detector diffractometer	1591 independent reflections
Radiation source: fine-focus sealed tube	1265 reflections with *I* > 2σ(*I*)
graphite	*R*_int_ = 0.026
ω scans	θ_max_ = 27.0°, θ_min_ = 1.6°
Absorption correction: multi-scan (*SADABS*; Bruker, 2001)	*h* = −10→9
*T*_min_ = 0.980, *T*_max_ = 0.982	*k* = −8→8
4175 measured reflections	*l* = −16→14

### Refinement

**Table:**

Refinement on *F*^2^	Primary atom site location: structure-invariant direct methods
Least-squares matrix: full	Secondary atom site location: difference Fourier map
*R*[*F*^2^ > 2σ(*F*^2^)] = 0.039	Hydrogen site location: inferred from neighbouring sites
*wR*(*F*^2^) = 0.105	H atoms treated by a mixture of independent and constrained refinement
*S* = 1.04	*w* = 1/[σ^2^(*F*_o_^2^) + (0.0566*P*)^2^ + 0.0371*P*] where *P* = (*F*_o_^2^ + 2*F*_c_^2^)/3
1591 reflections	(Δ/σ)_max_ = 0.001
191 parameters	Δρ_max_ = 0.12 e Å^−^^3^
5 restraints	Δρ_min_ = −0.17 e Å^−^^3^

### Special details

**Table:**

Geometry. All e.s.d.'s (except the e.s.d. in the dihedral angle between two l.s. planes) are estimated using the full covariance matrix. The cell e.s.d.'s are taken into account individually in the estimation of e.s.d.'s in distances, angles and torsion angles; correlations between e.s.d.'s in cell parameters are only used when they are defined by crystal symmetry. An approximate (isotropic) treatment of cell e.s.d.'s is used for estimating e.s.d.'s involving l.s. planes.
Refinement. Refinement of *F*^2^ against ALL reflections. The weighted *R*-factor *wR* and goodness of fit *S* are based on *F*^2^, conventional *R*-factors *R* are based on *F*, with *F* set to zero for negative *F*^2^. The threshold expression of *F*^2^ > σ(*F*^2^) is used only for calculating *R*-factors(gt) *etc*. and is not relevant to the choice of reflections for refinement. *R*-factors based on *F*^2^ are statistically about twice as large as those based on *F*, and *R*- factors based on ALL data will be even larger.

### Fractional atomic coordinates and isotropic or equivalent isotropic displacement parameters (Å^2^)

**Table:**

	*x*	*y*	*z*	*U*_iso_*/*U*_eq_	
N1	0.6188 (2)	0.2067 (4)	0.26835 (16)	0.0512 (5)	
N2	0.5065 (3)	0.1703 (4)	0.08960 (18)	0.0686 (7)	
N3	1.1646 (3)	0.0460 (4)	0.2380 (2)	0.0627 (6)	
O1	1.1579 (3)	−0.0626 (4)	0.16070 (19)	0.0851 (7)	
O2	1.2502 (3)	0.0106 (4)	0.32683 (18)	0.0880 (7)	
O3	0.7086 (2)	0.8232 (3)	0.09467 (14)	0.0688 (5)	
O4	0.8038 (2)	0.8810 (3)	0.26794 (14)	0.0657 (5)	
C1	1.0666 (3)	0.2328 (4)	0.2239 (2)	0.0516 (6)	
C2	0.9742 (3)	0.2807 (4)	0.1239 (2)	0.0561 (6)	
H2	0.9713	0.1955	0.0661	0.067*	
C3	0.8860 (3)	0.4578 (4)	0.11130 (19)	0.0533 (6)	
H3	0.8237	0.4927	0.0441	0.064*	
C4	0.8890 (3)	0.5844 (4)	0.19746 (17)	0.0466 (5)	
C5	0.9823 (3)	0.5302 (4)	0.29720 (19)	0.0582 (7)	
H5	0.9845	0.6137	0.3556	0.070*	
C6	1.0720 (3)	0.3536 (4)	0.3108 (2)	0.0583 (7)	
H6	1.1348	0.3177	0.3777	0.070*	
C7	0.7922 (3)	0.7779 (4)	0.1853 (2)	0.0514 (6)	
C8	0.6412 (3)	0.3019 (5)	0.3640 (2)	0.0608 (7)	
C9	0.5624 (4)	0.4787 (5)	0.3702 (3)	0.0767 (9)	
H9	0.5770	0.5468	0.4350	0.092*	
C10	0.4597 (4)	0.5562 (5)	0.2784 (3)	0.0770 (9)	
H10	0.4059	0.6775	0.2823	0.092*	
C11	0.4361 (3)	0.4599 (5)	0.1837 (3)	0.0656 (8)	
H11	0.3662	0.5135	0.1231	0.079*	
C12	0.5192 (3)	0.2767 (4)	0.1779 (2)	0.0542 (6)	
C13	0.7530 (4)	0.2001 (7)	0.4557 (2)	0.0845 (10)	
H13A	0.7053	0.0731	0.4672	0.127*	
H13B	0.7622	0.2801	0.5190	0.127*	
H13C	0.8636	0.1816	0.4399	0.127*	
H1	0.676 (4)	0.092 (3)	0.264 (2)	0.080*	
H2A	0.440 (3)	0.204 (5)	0.0282 (13)	0.080*	
H2B	0.571 (3)	0.064 (3)	0.088 (2)	0.080*	

### Atomic displacement parameters (Å^2^)

**Table:**

	*U*^11^	*U*^22^	*U*^33^	*U*^12^	*U*^13^	*U*^23^
N1	0.0508 (11)	0.0513 (13)	0.0501 (10)	0.0044 (10)	0.0069 (8)	0.0074 (10)
N2	0.0733 (15)	0.0610 (16)	0.0586 (13)	0.0073 (13)	−0.0163 (11)	0.0064 (13)
N3	0.0631 (13)	0.0528 (15)	0.0738 (15)	0.0060 (11)	0.0178 (11)	0.0139 (13)
O1	0.1048 (16)	0.0672 (14)	0.0887 (15)	0.0245 (13)	0.0325 (13)	0.0011 (13)
O2	0.0926 (15)	0.0715 (16)	0.0917 (14)	0.0277 (13)	−0.0003 (12)	0.0160 (13)
O3	0.0837 (12)	0.0484 (11)	0.0595 (10)	0.0035 (10)	−0.0198 (8)	0.0002 (9)
O4	0.0827 (12)	0.0477 (11)	0.0568 (10)	0.0119 (10)	−0.0088 (9)	−0.0042 (9)
C1	0.0492 (12)	0.0439 (14)	0.0621 (14)	−0.0003 (10)	0.0125 (10)	0.0091 (12)
C2	0.0641 (14)	0.0527 (16)	0.0516 (13)	0.0013 (13)	0.0118 (11)	−0.0010 (12)
C3	0.0603 (14)	0.0508 (15)	0.0449 (12)	−0.0013 (12)	0.0019 (11)	0.0042 (12)
C4	0.0483 (12)	0.0389 (12)	0.0485 (12)	−0.0047 (10)	0.0001 (9)	0.0038 (10)
C5	0.0675 (16)	0.0501 (15)	0.0502 (13)	0.0024 (13)	−0.0042 (11)	−0.0028 (13)
C6	0.0625 (15)	0.0531 (17)	0.0531 (13)	0.0044 (12)	−0.0027 (11)	0.0072 (12)
C7	0.0535 (12)	0.0394 (13)	0.0539 (13)	−0.0048 (11)	−0.0062 (10)	0.0004 (12)
C8	0.0603 (14)	0.0689 (17)	0.0555 (14)	0.0064 (14)	0.0173 (11)	0.0015 (14)
C9	0.079 (2)	0.078 (2)	0.0775 (19)	0.0153 (18)	0.0268 (16)	−0.0056 (18)
C10	0.0680 (17)	0.066 (2)	0.102 (2)	0.0169 (16)	0.0304 (16)	0.004 (2)
C11	0.0519 (14)	0.0621 (18)	0.0810 (19)	0.0102 (13)	0.0097 (13)	0.0150 (16)
C12	0.0457 (12)	0.0548 (16)	0.0589 (14)	−0.0025 (11)	0.0033 (10)	0.0115 (13)
C13	0.099 (2)	0.102 (3)	0.0512 (15)	0.022 (2)	0.0107 (14)	0.0011 (18)

### Geometric parameters (Å, °)

**Table:**

N1—C12	1.348 (3)	C4—C5	1.386 (3)
N1—C8	1.356 (3)	C4—C7	1.508 (3)
N1—H1	0.908 (10)	C5—C6	1.382 (4)
N2—C12	1.320 (4)	C5—H5	0.9300
N2—H2A	0.883 (10)	C6—H6	0.9300
N2—H2B	0.889 (10)	C8—C9	1.357 (4)
N3—O1	1.218 (3)	C8—C13	1.488 (4)
N3—O2	1.223 (3)	C9—C10	1.389 (4)
N3—C1	1.475 (3)	C9—H9	0.9300
O3—C7	1.250 (3)	C10—C11	1.349 (4)
O4—C7	1.249 (3)	C10—H10	0.9300
C1—C6	1.368 (4)	C11—C12	1.411 (4)
C1—C2	1.376 (3)	C11—H11	0.9300
C2—C3	1.379 (4)	C13—H13A	0.9600
C2—H2	0.9300	C13—H13B	0.9600
C3—C4	1.386 (3)	C13—H13C	0.9600
C3—H3	0.9300		
			
C12—N1—C8	123.4 (2)	C5—C6—H6	120.7
C12—N1—H1	117.8 (19)	O4—C7—O3	125.3 (2)
C8—N1—H1	118.7 (19)	O4—C7—C4	116.4 (2)
C12—N2—H2A	122.9 (19)	O3—C7—C4	118.3 (2)
C12—N2—H2B	121.1 (18)	N1—C8—C9	119.2 (3)
H2A—N2—H2B	116 (2)	N1—C8—C13	115.9 (3)
O1—N3—O2	123.8 (2)	C9—C8—C13	124.9 (3)
O1—N3—C1	118.5 (2)	C8—C9—C10	118.9 (3)
O2—N3—C1	117.7 (2)	C8—C9—H9	120.5
C6—C1—C2	122.2 (2)	C10—C9—H9	120.5
C6—C1—N3	118.7 (2)	C11—C10—C9	121.7 (3)
C2—C1—N3	119.1 (2)	C11—C10—H10	119.2
C1—C2—C3	118.5 (2)	C9—C10—H10	119.2
C1—C2—H2	120.7	C10—C11—C12	119.0 (3)
C3—C2—H2	120.7	C10—C11—H11	120.5
C2—C3—C4	120.9 (2)	C12—C11—H11	120.5
C2—C3—H3	119.6	N2—C12—N1	118.0 (2)
C4—C3—H3	119.6	N2—C12—C11	124.3 (2)
C3—C4—C5	118.9 (2)	N1—C12—C11	117.7 (3)
C3—C4—C7	121.6 (2)	C8—C13—H13A	109.5
C5—C4—C7	119.5 (2)	C8—C13—H13B	109.5
C6—C5—C4	120.8 (2)	H13A—C13—H13B	109.5
C6—C5—H5	119.6	C8—C13—H13C	109.5
C4—C5—H5	119.6	H13A—C13—H13C	109.5
C1—C6—C5	118.6 (2)	H13B—C13—H13C	109.5
C1—C6—H6	120.7		

### Hydrogen-bond geometry (Å, °)

**Table:**

*D*—H···*A*	*D*—H	H···*A*	*D*···*A*	*D*—H···*A*
N1—H1···O4^i^	0.91 (2)	1.75 (3)	2.649 (3)	173 (2)
N2—H2A···O3^ii^	0.89 (2)	1.94 (2)	2.812 (3)	170.(2)
N2—H2B···O3^i^	0.89 (2)	1.95 (2)	2.838 (3)	176 (2)

Symmetry codes: (i) *x*, *y*−1, *z*; (ii) −*x*+1, *y*−1/2, −*z*.
